# Research involving adults lacking capacity to consent: a content analysis of participant information sheets for consultees and legal representatives in England and Wales

**DOI:** 10.1186/s13063-019-3340-5

**Published:** 2019-04-25

**Authors:** Victoria Shepherd, Fiona Wood, Richard Griffith, Mark Sheehan, Kerenza Hood

**Affiliations:** 1 Division of Population Medicine, Cardiff University, Heath Park, Cardiff, CF14 4YS UK; 2 Centre for Trials Research, Cardiff University, Heath Park, Cardiff, CF14 4YS UK; 3 College of Human and Health Studies, Swansea University, Singleton Park, Swansea, SA2 8PP UK; 4 Ethox Centre, University of Oxford, Big Data Institute, Old Road Campus, Oxford, OX3 7LF UK

**Keywords:** Informed consent, mental capacity, proxy, content analysis, participant information sheets, randomised controlled trials

## Abstract

**Background:**

Research involving adults who lack the capacity to provide informed consent can be challenging. In England and Wales there are legal provisions for consulting with others who know the person with impaired capacity. The role of the ‘proxy’ (or ‘surrogate’) is to advise researchers about the person’s wishes and feelings or to provide consent on the person’s behalf for a clinical trial of a medicine. Information about the study is usually provided to the proxy; however, little information is available to proxies about their role, or the appropriate legal and ethical basis for their decision, to help inform their decision-making. The aim of this study was to analyse the written information that is provided to consultees and legal representatives.

**Methods:**

Studies including adults lacking capacity to consent which utilised consultees or legal representatives were identified using the UK Clinical Trials Gateway database. A representative sample (*n* = 30) were randomly selected. Information sheets and other study documents provided to proxies were obtained, and relevant content was extracted. Content analysis was conducted through four stages: decontextualisation of the unit of analysis, recontextualisation, categorisation, and compilation. The data were summarised narratively according to each theme and category.

**Results:**

Considerable variation was found in the written information sheets provided to proxies. Most directed proxies to consider the wishes and feelings of the person who lacked capacity and to consult with others during the decision-making process. However, a small number of studies extended the scope of the proxy’s role to consider the person’s suitability or eligibility for the study. Particular discrepancies were found in information provided to those acting as consultees or legal representatives in a professional, as opposed to a personal, capacity. Incorrect uses of terminology were frequently found, and a small number of studies inaccurately interpreted the law.

**Conclusions:**

Despite undergoing ethical review, study documents lacked essential information, incorrectly used terminology, and conflated professionals’ clinical and representation roles. Future recommendations include ensuring proxies are provided with adequate and accurate information which complies with the legal frameworks. Further research is needed to explore the information and decision-making needs of those acting as consultees and legal representatives.

**Electronic supplementary material:**

The online version of this article (10.1186/s13063-019-3340-5) contains supplementary material, which is available to authorized users.

## Background

Informed consent is fundamental to ethically conducted medical research [1], but obtaining valid consent can be particularly challenging in specific practice contexts [2]. Individuals may be unable to provide consent for themselves due to an impairing medical condition that strikes suddenly (like a stroke) or causes a gradual loss of capacity (such as dementia) or due to profound learning disabilities. The exclusion of those who lack mental capacity from participating in research has been highlighted as a concern [3], as it results in a lack of evidence-based care for populations who may already experience significant health disparities [4].

In England and Wales the Mental Capacity Act 2005 (MCA) has provision for consulting an individual who knows the person with impaired capacity well, such as a family member, to advise about research participation on the person’s behalf [5]. The consultee is provided with information about the project and asked what the potential participant’s likely wishes and feelings would be about taking part in the project if he or she had capacity [5]. Any indication that the person would not have wished to participate must be respected [5]. Responsibility for deciding whether to include a person lacking capacity lies ultimately with the researcher [5]. Clinical trials of investigational medicinal products (CTIMPs) are regulated separately in the UK under the Medicines for Human Use (Clinical Trials) Regulations 2004 (CTR) [6], although this is shortly to be replaced with the Clinical Trials Regulation No 536/2014 [7]. Under the CTR, the relative or friend acting as legal representative must decide whether the person lacking capacity should participate in the trial on the basis of what they would have wanted had they the capacity to choose for themselves, their ‘presumed will’ [6]. The legal representative must be given the opportunity to understand the objectives, risks, and inconveniences of the trial, and then provides informed consent on behalf of the person who lacks capacity [6]. Should no appropriate relative or friend be available or willing to act as the person’s ‘proxy’ or ‘surrogate’, under both the MCA and CTR there are provisions for a professional to act as a nominated consultee (s32(2)) [5, 8] or legal representative (Schedule 1, Part 1(2(a)(ii))) [6]. The term proxy is used in this paper to include both consultees and legal representatives acting in either a personal or professional capacity.

Written information about the study is tailored for proxies, usually in the form of an amended version of the information provided to participants themselves. If a person is willing to act as proxy, he/she will then be asked to provide written confirmation of his/her advice regarding the person’s wishes [5] or informed consent on that person’s behalf [6] using a consent or declaration form. Proxy versions of participant information sheets (PISs) and consent or declaration forms are not standardised, and there is minimal guidance available for researchers when drafting documents for studies involving adults lacking capacity, although templates are available [9]. Neither the MCA nor the CTR have requirements regarding the information that should be given to the person acting as proxy about his/her role, only concerning information about the project [5] or the objectives, risks, and inconveniences of the trial and the conditions under which it is to be conducted [6]. There is no guidance for proxies about how their decision should be made or what to do if they are unable to determine what the person would have wanted. Decision-making may be challenging for proxies. Preferences regarding future participation in research are rarely discussed and, for individuals who have never held relevant views and preferences, they will be impossible to determine. Proxies are acutely aware of the moral difference between deciding for oneself and deciding for others. They report that decisions about research participation are burdensome [10], and they experience varying degrees of comfort and confidence in proxy decision-making. Many studies have empirically evaluated the readability and content of PISs and consent forms [11–15] and assessed participant comprehension [16]. However, no studies have examined written information provided to proxies.

Documents can be considered as socially situated products which are produced, consumed, and used in organised settings, and understanding how they function is important [17]. Content analysis is an empirically grounded research method [18] which has been described as providing a ‘systematic and objective means to make valid inferences from verbal, visual, or written data in order to describe and quantify specific phenomena’ (p. 314) [18]. Content analysis has been used in a range of academic disciplines, including social and healthcare sciences, and has been applied to a variety of data and to various depths of interpretation, using both qualitative and quantitative methodology [19].

This study aimed to explore the written information provided to proxies who have been approached to be involved in decision-making about research participation for an adult who lacks the capacity to provide his/her own consent. The information that related specifically to the proxy’s role was reviewed, rather than information about the study per se. Attention was paid to the context in which the documents were used: the type of research and study population, information about why the proxy had been approached (including whether they were approached in a personal or professional capacity), the proxy’s role in the decision-making process, the required basis for the proxy’s decision, and any information or guidance about how the proxy might approach the decision including any sources of guidance or support. The correct legal basis was determined by reference to the legislation governing research involving adults lacking capacity in England and Wales, the MCA [5], and CTR [6].

## Methods

### Sampling

Current or recently completed (within the preceding 3 years) studies were identified from the National Institute for Health Research (NIHR) co-ordinated UK Clinical Trials Gateway (UKCTG) public database (formerly the NIHR Portfolio database). Studies which included participants aged 16+ years who may lack decision-making capacity, and therefore require proxy involvement, were eligible for inclusion. Whilst capacity is considered decision-specific rather than global and is not a static construct [5], the circumstances under which a proxy decision-maker is required and the proxy’s prior experience of acting as decision-maker are relevant factors in this context. The UKCTG is primarily intended for patients to find relevant clinical trials to participate in, and so the database uses condition-specific search terms, in addition to filters such as trial status. Therefore studies which included adults who lacked capacity were identified by searching the database for appropriate medical conditions or populations which are more likely to be associated with cognitive impairment. Studies which involved emergency research, and therefore a consent waiver, were excluded.

A search strategy was designed to include trials conducted in different decisional contexts through classifying the circumstances under which proxy involvement is required, either as part of a progressive process, following a sudden or acute event, or to reflect long-term circumstances. This necessitated a pragmatic search strategy which identified condition-specific search terms (as required by the UKCTG) that would capture trials across these three areas. It is recognised that a lack of capacity cannot be established by reference to a condition [5], and only some individuals living with these conditions will experience any cognitive impairment or impaired capacity specific to the particular enrolment decision. Searches were conducted in June and July 2017 by one researcher (VS) using search terms agreed by three researchers (VS, FW, KH). Search terms to identify eligible studies were divided between three groups of decisional contexts:

- Progressive process: search terms ‘dementia’ (all types), ‘Huntington’s Disease’
- Sudden or acute event: search terms ‘stroke’, ‘traumatic brain injury’, ‘critical care’
- Long-term circumstances: search terms ‘Down’s syndrome’, ‘intellectual disability’, ‘learning disability’

All types of research designs were included. A sample of 30 studies was randomly selected from the list of eligible studies, stratified by the three groups of decisional context. In content analysis, there are no established criteria for the number of sampling units or objects to study; the sample size is based on the informational needs and the ability to answer the research question with confidence [20]. The sample size estimation for this analysis was derived from a similar study examining consent forms for clinical genetic content [15].

All study documents provided to the proxy were obtained through the UKCTG database links (funder or sponsor’s website, study website, etc.) or were requested from the lead investigator, project co-ordinator, or sponsor as appropriate. Studies were only eligible if study documents were available. Where study documents could not be obtained, the study was considered to be ineligible, and a replacement study was randomly selected from the same condition/group. Sampling continued until the target sample was reached.

### Data collection

The sampling unit for inclusion was the study; the unit of analysis was the documents provided to proxies to provide information to help inform their decision-making for each study. These documents included proxy/participant information sheets, informed consent or declaration forms, or other relevant documents. Studies were allocated a unique reference number and anonymised to remove any identifiable information. Study documents were reviewed and analysed for content relating to the role of the proxy and the decision about research participation, and the area of interest data extracted. The respective numbers and types of documents were recorded by individual study and per group (progressive loss of capacity, acute loss of capacity, no prior capacity).

### Content analysis

Content analysis can take many forms [17, 19]. This study was conducted using a pragmatic content analysis approach [17], incorporating both quantitative and qualitative analyses. Content areas, defined as parts of the text that address a specific topic, were identified and extracted. Content was divided into that which informed the proxy as to why they had been approached, the basis for their decisions, how the proxy might approach making a decision, practical instructions to be followed, and information about withdrawal from the study. The analysis process followed that outlined by Bengtsson [20] as a series of iterative steps, with the four main stages being decontextualisation of the unit of analysis, recontextualisation, categorisation, and compilation. The meaning unit (or coding or content unit) was defined as words, sentences, or paragraphs containing aspects related to each other through their content and context [19].

The documents were reviewed in order to ensure familiarity with the text, and the content areas from the study documents were extracted and entered into qualitative data analysis software (NVivo 11). During the decontextualisation stage the meaning unit (the words or sentences that are intended to convey an item of information or instruction to the proxy about their role or decision) was coded using a coding framework agreed between three researchers (VS, FW, KH). The data were coded iteratively by one researcher (VS), with discussion with the research team to increase the stability and reliability of the coding process. Sample data extracts were regularly reviewed by the group to ensure consistency of coding.

During the recontextualisation stage, the meaning units were re-read alongside the original data to ensure the content was adequately captured, with no extraneous data included that were not relevant to the aim of the study [20]. For the categorisation stage, the themes and categories emerging from the meaning units were identified. There are no universally adopted concepts for the headings used in content analysis [20]; however, themes were the broader overall concepts, and the categories were the smaller sub-themes that brought together a number of related meaning units.

The compilation stage drew on a manifest level of analysis, which stays very close to the original text to describe *what was said* using the visible and obvious [21]. Given the nature of data contained in these types of documents, a manifest analysis which stays closer to the original meaning and context was considered to be appropriate. The data were summarised narratively according to each theme and category. A summary of themes and categories was tabulated, with illustrative meaning units presented. The themes and categories were quantified to allow a greater representation of information [20].

## Results

Of 1194 potentially eligible studies identified, 70 studies (6%) included adults lacking capacity. Study documents could not be obtained for 15 studies, because either there were no viable contact details for the study or there was no response from the study team. There were no refusals. Sampling ceased when documents had been obtained for a total of 30 studies, which were subsequently included in the review (Table 1). An additional file shows the study characteristics in more detail (Additional file 1). Studies included both observational and interventional studies, of which 9 (30%) were classified as a CTIMP. The majority of studies were sponsored either by a higher education institute (*n* = 19, 63%) or by a National Health Service (NHS) organisation (*n* = 10, 33%). The NIHR was the funding body in 22 studies (73%), followed by charitable funders (*n* = 7, 23%). Studies were either currently ongoing (*n* = 14, 47%) or had been completed within the previous 3 years (*n* = 16, 53%).

**Table 1:** Characteristics of screened and included studies by decisional context

Decisional context	Search term/condition^a^	No. studies identified	No. potentially eligible^b^ studies	No. studies included	No. CTIMPs^c^ included	Study IDs
Long-term circumstances	Down’s syndrome	181	9	4	1	01–04
	Intellectual disability					
	Learning disability					
Progressive process	Alzheimer’s disease	505	33	12	1	05–16
	Dementia					
	Huntingdon’s disease					
	Care home(s)					
Sudden/acute event	Critical care	508	28	14	7	17–30
	Acute stroke					
	Traumatic brain injury					
Total		1194	70	30	9	

^a^Search terms are filters or categories used by the UKCTG to enable users to search for clinical trials by condition or disease area

^b^Eligible if study documents are available (all other eligibility criteria having been met)

^c^Number of clinical trials of investigational medicinal products (CTIMPs) included

Studies primarily combined information about the proxy’s role with information about the study itself into a single document (*n* = 28, 93%), although two studies had separate documents for the role of the proxy and the study information which was contained in the standard PIS (study ID 04, ID 12). Consequently, study information sheets ranged considerably in length (Table 2). Where the information sheet combined information about the proxy’s role and the study, the area of interest that related to the proxy’s role (area of interest/total length of document) comprised 7–68% of the total length of the document.

**Table 2:** Quantitative data from study information sheets

	Total no. of documents *n* = 42 (*%*)	Unit size by no. words range (*median*)
Total length of document	42	217–3997 (1665)
CTIMP	14	217–2676 (2067)
Non-CTIMP	28	230–3997 (1602)
Total area of interest	42	79–926 (344)
Proportion of total length of document (area of interest/total length of document)		79/1197–641/953 7–68%
CTIMP	14	79–371 (232)
Non-CTIMP	28	155–926 (422)
Why the proxy is being approached	42 (100%)	22–494 (104)
CTIMP	14	22–217 (122)
Non-CTIMP	28	27–494 (98)
Basis for the decision	38 (90%)	8–267 (83)
CTIMP	10	14–123 (51)
Non-CTIMP	28	8–267 (107)
How the proxy might approach deciding	13 (31%)	18–185 (67)
CTIMP	1	76
Non-CTIMP	12	18–185 (66)
Practical instructions for the proxy	38 (90%)	18–309 (80)
CTIMP	10	18–151 (75)
Non-CTIMP	28	28–309 (83)
Withdrawal from the study (including proxy’s role)	34 (81%)	19–168 (44)
CTIMP	11	25–87 (49)
Non-CTIMP	23	19–168 (43)

*CTIMP* Clinical trial of an investigational medicinal product, governed by CTR

*Non-CTIMP* Research other than a clinical trial of an investigational medicinal product, governed by MCA

Studies were divided into those that provided separate documents for proxies acting in a personal capacity (relative or friend as a personal consultee or personal legal representative) and a professional capacity (member of the care team as a nominated consultee or professional legal representative) (*n* = 10, 33%), those with joint documents for both personal and professional proxies (*n* = 9, 30%), studies that provided documents for personal proxies only (*n* = 8, 27%), or where it was assumed that there was no provision for professional proxies as information sheets were not provided for professional proxies (*n* = 3, 10%).

Content relating to why the proxy was being approached was found in all 42 study documents. Content relating to other categories of information varied considerably, with only 13 documents (31%) providing information about how the proxy might approach decision-making (Table 3).

**Table 3:** Content examples by category

Why the proxy is being approached	
We would like to invite your relative/friend to be part of a research study. We feel that he/she may not be able to decide for himself/herself whether to participate in this research. We would therefore like to ask your opinion as to whether or not you think he/she would want to be involved (*personal consultee information sheet ID 214*) We would like to invite one of your residents to take part in our research study. We feel that they are unable to decide for themselves whether to participate in this research. To help decide if he/she should join the study, we would like to ask your opinion about whether you think they would want to be involved (*nominated consultee information sheet ID 218*) We are asking you, as a [title of medical role] or [title of medical role], to consider whether you will be able to act as a professional legal representative to provide agreement for your patient to participate in the [name of trial]. The majority of patients who will be eligible for this trial, due to the severity of their injuries, will not have capacity to provide informed consent for enrolment into the trial (*professional legal representative information sheet ID 306*)	
Basis for the decision	
You are being asked to advise the researchers about this person’s wishes and feelings as to whether they themselves would have wished to join this research (*personal consultee information sheet ID 105*) If you agree please read the information sheet carefully and give your opinion as to whether or not you think this patient would be willing to participate in this medical research (*professional legal representative information sheet ID 311*)	
How the proxy might approach deciding	
Using what you know of the wishes and feelings of your relative/friend, please advise us on whether you feel he/she would have agreed to join the study, if he/she had been able to decide for him/herself. Please base your advice on your knowledge of your relative/friend and their past and present views or feelings, not on your own views of research in general or this project. You should try to seek the views of your relative/friend, if appropriate, and also the views of other family or friends in helping give this advice (*personal consultee information sheet ID 213*) If you have known them for some time, you may be aware of any views they may have about taking part in such a project or whether they have made an ‘Advance Decision’. If the potential participant has made an ‘Advance Decision’ this is important, as it shows that they have already made decisions for themselves (*professional legal representative information sheet ID 105*) Think about the broad aims of the research, the risks and benefits, and what taking part will mean for this person (*combined personal and nominated consultee information sheet ID 203*)	
Practical instructions for the proxy	
If you are prepared to act as the consultee you will be provided with a copy of the participant information sheet and be given an opportunity to discuss the project with one of the researchers so that you can form an opinion as to the individual’s likely wishes/feelings with respect to the project. If, at the end of this process, you feel that the individual would like to take part in the project, you will be asked to sign a form to that effect (*personal consultee information sheet ID 108*) Please take time to read the following information carefully. Talk to others about the study if you wish. Ask us if there is anything that is not clear to you, or if you would like more information. Take time to decide whether or not you wish the patient to take part. If you do decide for them to take part, you will be given this information sheet to keep and be asked to sign a consent form (*professional legal representative information sheet ID 316*)	
Withdrawal from the study	
If the resident does take part in the research, we will keep you fully informed during the study so you can let us know if you have any concerns or you think the resident no longer wishes to take part (*nominated consultee information sheet ID 214*) If you later decide that he/she no longer wishes to take part, please inform us and he/she will be withdrawn from the study. You do not need to give a reason and it will not affect the standard of care your relative receives (*personal consultee information sheet ID 317*)	

### Key themes

#### Representing the wishes, feelings, and interests of the person with impaired capacity

Almost all studies advised or directed proxies to consider the wishes and feelings using standard phrases such as ‘You are being asked to advise the researchers about this person’s wishes and feelings’. Some extended this to advising on the person’s views about taking part in research in general or the particular study in question, using phrases such as ‘you may be aware of any views they may have about taking part in such a project’.

With the exception of two, studies made no reference to the temporal aspects of the person’s wishes and feelings — whether the proxy should consider their past or presently expressed wishes, and the comparative weight that should be afforded to current and prior wishes should they conflict. The two study documents that did include temporal considerations stated that proxies should base their advice on their knowledge of their friend/relative and both ‘their past and present views or feelings’ (ID 12, ID 14). The MCA uses the future conditional tense to require the proxy to consider ‘what the person’s wishes and feelings about taking part in the project *would be likely to be* if they had capacity in relation to the matter’ (s32(4b)) [5]. The CTR provide less guidance, merely that the informed consent given ‘shall represent that adult’s presumed will’ (Schedule 1, Part 5, Principle 12) [6]. Some study documents extended the proxy’s role to consider the interests, as well as the wishes and feelings, of the person, which reflects the phrasing used in the template developed by the UK Health Research Authority (HRA) [7]. One study listed the following factors the proxy should consider: the aims of the research, previous thoughts and wishes, risks and benefits to the patient and others, how being involved with the research would affect the patient’s routine, and any advance decisions about participating in research the patient may have made (ID 15). One study advised the professional representative to ensure that there were no other known factors (e.g. cultural or religious beliefs) which may influence whether the patient would want to participate in medical research (ID 19).

Some study documents directed which perspective or viewpoint the proxy was expected to take, by advising the proxy to disregard his/her own views when making a decision: ‘it is important that you should set aside any of your own personal views about the project’ or ‘your views of research in general’ (ID 02, ID 06, ID 12, ID 14, ID 22).

#### Consulting with others

A number of the study documents (*n* = 16) included advising or directing the proxy to consult with others during the decision-making process, phrased as either talking to others if wished or if necessary. Some specified whom might be consulted: other relatives, friends, or healthcare professionals, although professionals were advised to restrict this to ‘other colleagues who have an interest in the person’s welfare, but who won’t be involved in the research themselves’ (ID 12). Although there is no specific requirement to consult others under either the MCA or CTR, the general principle of the MCA is to consult others who are engaged in caring for the person or interested in the person’s welfare when decisions are made about research participation (s4(7b)) [5]. If the proxy was uncertain about taking on the role, eight studies advised that he/she could seek independent advice, although no information was provided about how or where that advice could be obtained. A small number advised the proxy to consult with the person themselves, phrased as to ‘attempt’ or ‘try to’ seek the views of the person. These were all from studies involving care home residents (ID 12, ID 13, ID 14).

#### Consultee’s role

Some of the non-CTIMP studies made it clear that the consultee was not being asked to provide consent on the person’s behalf, as would be the case if they were a legal representative in a CTIMP. This was either explicitly stated in the description of the proxy’s role: ‘A personal consultee is not asked to provide consent for or on behalf of their relative/friend’ (ID 12) or implicit in the description of the proxy’s role being to ‘give advice’ about the person’s wishes (ID 04). In some documents where this statement was included, the wrong terminology was sometimes still used: ‘After appropriate consultation we would like you to complete the attached *consent form* indicating whether you feel the named person would or would not have wished to participate’ (ID 14), and ‘The consultee does not give consent, only advice’ followed by ‘We would also like to seek your consent so that any remaining samples may be stored and used in possible future research’ (ID 28) although this is not required [22]. Only one non-CTIMP study directly stated that the responsibility to decide whether the participant should be entered into the research lies ultimately with the researcher (ID 16).

#### Extension of proxy’s role to include determining eligibility

A small number of studies appeared to extend the scope of the proxy’s role beyond that of representing the person’s wishes or feelings about participation, or their presumed will. One document provided to personal consultees had the option for the consultee to decline the study on behalf of the person if they did not consider that their relative/friend was ‘well enough to take part’ (ID 12). The role of professionals acting as legal representative was sometimes extended to determining the person’s eligibility for the study, either explicitly (ID 19, ID 27) or implicitly by confirming that they understand ‘what the study involves, including inclusion and exclusion criteria’ (ID 30). Eligibility forms part of the investigator’s role; the role of the professional legal representative is to represent the person’s wishes and feelings as someone who is unconnected to the study.

#### Advance decisions

Some studies asked proxies to inform the researchers about any advance decisions the person may have made, as these ‘should take’ or ‘will take’ precedence (ID 02, ID 25). Others extended the scope to ‘any advance decisions they may have made about participating in research’ (ID 11, ID 13, ID 15, ID 22, ID 26), which reflects the wording used in the HRA template [9]. The MCA has provisions for Advance Decisions to Refuse Treatment (ADRT) which relate to *refusal* rather than a positive request, and it refers to *medical treatment decisions* rather than those about research [5]. The MCA Code of Practice states ‘Researchers must not do anything the person who lacks capacity objects to. They must not do anything to go against any advance decision to refuse treatment or other statement the person has previously made expressing preferences about their care or treatment’ (s11.30) [23]. This means the researcher has an obligation to respect the person’s expression of refusal that may conflict with participation in a research study, where the care/treatment is part of the intervention or associated requirements.

#### Inaccurate use of terminology

There were many instances of confusion in the use of the terms for the proxy, where the proxy was called a ‘legal representative’ when the study did not fall under the scope of the CTR and therefore the proper term should have been ‘consultee’, or the term ‘consultee’ was used when the MCA was not the governing legislation and the proxy was therefore acting as a legal representative. A combination of terms was used for proxies in some studies (‘professional consultee’ ID 17), and alternative terms were introduced in some studies: ‘independent physician’ and ‘proxy relative’ (ID 27) and ‘Registered Medical Practitioner’ (ID 30). The term ‘assent’ was used in three studies (ID 12, ID 14, ID 22) either in the information sheet provided to the proxy or in the title of the document. Assent is not a recognised term in legislation governing research involving adults in England and Wales, although it is a legally recognised term in paediatric research, and is used informally by some research professionals when referring to the involvement of adults who lack capacity to consent.

The term ‘consent’ was used when the process was in fact consultation (ID 18, ID 22) or where the consultee declaration form required the ‘Name of Person taking consent’ (ID 03). The opposite was also observed, where informed consent should have been obtained from the legal representative, but the documents referred to ‘consultation’ and ‘consultee’ (ID 26) or ‘declaration’ as illustrated by ‘We will seek written informed consent from the patient, or declaration from a personal legal representative, as soon as possible after the patient’s admission’ (ID 19).

#### Disconnect between information provided to professional and personal proxies

Not all studies included the option of a professional acting as proxy, and it was not clear whether the person could take part in the study if no personal proxy was able or willing to be involved. In spite of the shared legal basis of personal and professional proxies, some studies appeared to differentiate between them. This extended to consent forms for two clinical trials that both visually and in terms of content differed significantly from standard consent forms used in research, where the appearance was consistent with a letter and did not contain individual statements or boxes to be initialled, and no counter signature was required from the person obtaining consent (ID 09, ID 23). Unlike the personal legal representative consent forms, they did not include similar statements about providing consent for access to medical notes by the research sponsor or other representatives ((ID 09, ID 23) or consent to obtain a blood sample for analysis for the study and retention for future related studies (ID 09). For one study there was no information sheet for professional legal representatives, and in place of a consent form there was a small section to be completed on the baseline case report form (ID 24). In a second study there was also no information sheet for the nominated consultee, and the ‘Registered Medical Practitioner form’ did not mention ‘consultee’ or refer to advice regarding the person’s wishes and feelings and had only two statements, one of which was that they had no objection and were not aware of any objections to the participant being enrolled in the study (ID 30). One information sheet for professional legal representatives listed the inclusion and exclusion criteria as well as details about the dose of the medicinal product being investigated, its preparation and administration, and trial unblinding procedures, whereas the equivalent for a personal legal representative did not (ID 19).

#### Inaccurate interpretation of the law

Some study documents conflicted with the regulatory frameworks through inaccurate interpretation of the legislation. This included statements such as ‘When determining who is able to provide such consent, the Medicines for Human Use Regulations state that the individuals’ parents should always be approached first’ for a trial involving adults only (ID 01). The CTR do not specify which family members or friends, or in what hierarchical order, the researcher should approach to act as legal representative [6]. Another instance included the requirement that mental capacity assessment be undertaken on all potential participants, during which specific details needed to be recalled by the person (a care home resident) prior to being deemed to have capacity to provide informed consent for the study (ID 05). The MCA clearly states that a person must be assumed to have capacity unless it is established that they lack capacity; this is a key principle of the Act (s1(2)) [5]. Other studies incorrectly stated the legal basis for the proxy’s decision, such as they are required to ‘assess whether study enrolment is in the patient’s best interest’ (ID 19). Whilst the MCA has as one of the underlying principles that ‘An act done, or decision made, under this Act for or on behalf of a person who lacks capacity must be done, or made, in his best interests’ (s1(5)) [5], the exception to this is regarding decisions about research (s2.12) [23].

## Discussion

This study examined study documents provided to consultees and legal representatives who are involved in decisions about research participation on behalf of an adult who lacks capacity to consent. Although there are important conceptual differences between providing consent for ourselves and on behalf of another, the results from this study supported previous research that found that many study documents lack items deemed to be important for promoting high-quality decisions [12, 24]. A lack of information about the proxy’s role and the legal basis for his/her decision risks failing to meet the requirements for decisions about research participation to be fully informed, and may therefore threaten the ethical conduct of such research [25]. The database search in this study also confirmed findings from other studies that only very small numbers of studies include people who lack capacity [3, 26, 27].

All included studies could recruit participants both with and without capacity and had study documents for the participant themselves if appropriate (these documents were not included in the study documents analysed). The length of information sheets varied considerably. Although CTIMP information sheets were longer than those for non-CTIMP studies (median number of words 2067 compared to 1602), as found in other studies [28], the content that related to the role of the proxy in CTIMPs was nearly half that provided in non-CTIMPs (median number of words 232 compared to 422). Information sheets for professionals acting as proxy (median number of words 1610) were shorter than those for personal proxies (1698 words) and for professionals and personal proxies jointly (1788 words).

All studies had received Research Ethics Committee (REC) approval, and either they were currently recruiting participants or recruitment had been completed. Despite this, issues with incorrect terminology were common. This suggests that issues identified in a study which was conducted shortly after the introduction of the MCA, where the legal requirements for research involving incapacitated adults were not being consistently or correctly interpreted by researchers and RECs [29], are largely unchanged in the decade since. This lack of clarity may contribute to the confusion and lack of understanding about the legislation by health and social care professionals [30], leading to the exclusion from research of those who lack capacity [4]. The discrepancy between the legislatory requirements and the research practice as a result of inaccurate interpretation of the law is also significant, as it may affect the identification and involvement of the correct proxy in the decision-making process and interfere with the appropriate legal and ethical basis for that decision.

Information sheets varied considerably in the content relating to how the proxy should make a decision, with 21 studies not providing any such information across a total of 29 documents. Four of the information sheets did not provide information about withdrawing the person from participating, and a further two provided it to the personal proxy only and not the professional acting as proxy (ID 19, ID 23). These two studies involved the participant being followed up for 28 days. Requesting withdrawal is a key part of the proxy’s role throughout the whole duration of a participant’s involvement in a study (s32(5)) [5], although it was included as a statement in the consent form for one of the two studies (ID 19). Some studies did inform proxies that the researchers would seek consent from the participant once (or if) they regained capacity or from relatives/friends in the interim, but this risks leaving the patient without anyone to represent him/her in the intervening period. The few documents which advised that the patient should be involved in the decision were from studies being conducted with older people living in care homes. People living with dementia, which affects around 69% of care home residents [31, 32], may experience greater fluctuation and variation in decision-making capacity [33] compared to the populations included in other studies, although all adults lacking capacity should be supported and involved in decisions that concern them as far as is possible [23, 34]. Under the CTR, the person lacking capacity must have ‘received information according to his capacity of understanding regarding the trial, its risks and its benefits’ [6]. Although arguably this might form part of the researcher’s obligation, in general the study documents do not identify the need to inform the persons lacking capacity about the trial or to include them in the decision.

Disparities were seen between documents for professionals acting as proxy and those for relatives/friends, particularly in acute and critical care studies. The role of the professional representative was distinguished from that of the personal proxy in several of the studies, both in the amount and content of the information provided to them and in how their consent or advice was sought and documented. This role was ‘medicalised’ in some settings (notably in critical care studies), where it was treated more like a consultation for a second medical opinion about the patient’s suitability and eligibility for the study rather than an attempt to represent the person’s wishes and feelings about taking part. This resulted in a conflation of professionals’ clinical and representation roles, which may be due to the difficulty fulfilling the legal requirement for representing the ‘presumed will’ of a person who is unconscious or has significantly impaired capacity in an acute or critical care setting where no previous relationship exists between the healthcare professional and patient. This was in contrast to studies in care home settings, where the professional acting as proxy is likely to have developed a close relationship with the person in their care, allowing them to more fully represent the person’s current, if not past, wishes and feelings about being part of a research study. Studies consistently failed to address the complex issue of how the proxy would be able to represent the person’s wishes and feelings if such wishes had never been expressed. These may be intractable questions due to the nature of the requirements of the current legislation. There is currently no HRA information sheet template for clinical trials which fall under the CTR in the UK [9].

There appeared to be a disconnect between the conceptualisation of advance decisions under the MCA and the wording used in the documents, which was based on the HRA template information sheet [9]. This was seen both in terms of differences in scope, as the MCA provisions relate specifically to ADRTs) only [5], and because the negative orientation towards treatment options under the MCA means that an ADRT would be relevant only to studies involving *the treatment* that was being refused and not refusal *of research* in general. Only one study (ID 02) mentioned the role of a Lasting Power of Attorney (LPA) for health and welfare or a Court of Protection-appointed Deputyship who would be involved in decisions about care and treatment on behalf of a person with impaired capacity. However, the role of an LPA or Deputy in decisions about research remains unclear [35].

### Strengths and limitations

To the best of our knowledge, no previous studies have examined the information provided to proxies who are involved in decision-making about research participation by adults lacking capacity. Studies involving different populations and study designs were included in order to represent a range of contexts in which proxy decision-making occurs. Content analysis allowed a comprehensive understanding of both the study documents’ content and context.

The searches were conducted using one database which is not necessarily intended for searches of this nature; therefore, the search for eligible studies is by no means considered exhaustive. Studies could only be included where the documents were publicly available or where the investigators were willing to share the documents, and therefore selection bias may have been introduced. Documents could not be obtained for studies where the research team was uncontactable. This occurred because contact details were not listed on the UKCTG and could not be obtained using Internet searches, contact details were not operational, or there was no response from all identifiable study contacts. There were no refusals to provide study documents.

A further limitation is that a relatively small sample of studies was reviewed, although this represented 43% of the potentially eligible studies identified during the searches. Very small numbers of CTIMPs were found, and none of the included studies was sponsored or conducted by pharmaceutical companies. Most studies were publicly funded by the UK Department of Health and Social Care through the NIHR, the research arm of the NHS. Study populations did not include those participating in research involving end-of-life care or mental illness. The findings may not be representative of all study populations where participants may have impaired capacity. Study documents were limited to those used for participants in England and Wales only. Documents provided to participants in other areas of the UK (Scotland and Northern Ireland) will differ due to different legislation regarding research involving adults who lack capacity [36, 37], except clinical trials of medicinal products, which are regulated by UK-wide legislation [6].

An important limitation is that only written information was analysed in this study, which forms only one ‘piece of the jigsaw’ of decision-making [12]. In practice, information sheets are provided in addition to an interview with the research team, during which proxies will receive further information which may be tailored to their information and decision-making needs. Studies also used other forms of communicating with patients and proxies, such as providing brief or easy-read information to enhance the ability of the person with impaired capacity to understand study information and be supported to provide their own consent, or obtaining verbal consent where research was conducted following a medical emergency.

### Recommendations for future research

In-depth exploration of proxies’ information needs and decision-making processes may allow a greater understanding of how proxies are prepared for, and undertake, decision-making in practice. Observation of consultations with proxies, and proxies’ accounts of experiences of being consulted, including issues influencing their decisions, will provide additional information about proxy decision-making for research, which is currently poorly understood. This may assist in developing a minimum standard of information provision or evidence-based content which, if evaluated, would allow optimised written information to be provided by researchers. This would help ensure that proxies are fully informed about their role, enhance the consultation process with proxies, and support high-quality decision-making and the provision of truly informed consent where appropriate.

The development of interventions to inform and support those who design and conduct research studies which include adults lacking capacity, as well as those responsible for the ethical review of such studies, may be warranted.

### Recommendations for research practice

To improve comprehension and uncertainty for all those involved in research with adults lacking capacity, there is a need for accuracy in the use of terms for consultees and legal representatives by researchers in study documents. There needs to be greater clarity around the role of both personal and professional consultees and legal representatives, the basis for their decision, and whether they are being asked for consent or advice. Researchers may benefit from engaging with individuals or institutions with legal and ethical expertise when developing study documents involving populations where consent and recruitment may be complex. Consistency in the review of information sheets and consent/declaration forms by RECs could reduce the level of inaccuracy and, as a result, may reduce the confusion and misunderstanding for those either seeking or providing informed consent or advice for research participation.

To ensure compliance with the legal requirements, and following the principles of informed consent, information sheets should include sufficient information to allow proxies to understand why they are being approached and the basis on which they should make a decision. Orientating the proxy to making a decision based on what the patient himself would have decided, rather than the proxy’s own personal views about research in general or the particular study in question, may be of benefit. Ensuring the proxy is informed about his/her role in withdrawing the person from the study, should the proxy feel the person would wish to withdraw from it, should be clearly stated and arrangements made to ensure the person remains represented at all times. It is important that this does not contribute to an increase in the length of study documents. Using a ‘layered’ or ‘tiered’ approach to information provision can avoid overwhelming potential participants with lengthy and complicated PISs, whilst providing accurate and relevant information to ensure genuinely informed consent [38]. A ‘layered’ approach involves initially providing potential participants with a short summary including sufficient information needed to decide whether or not to take part in the research, with user-friendly methods of access to more detailed information presented in one or more additional layers [38]. The primary information should clearly explain how this further information may be accessed [38].

Further guidance for researchers when drafting documents for studies involving adults lacking capacity is recommended, particularly for clinical trials of medicinal products where template documents may be of benefit, and where professionals in acute and critical care settings are involved as consultees or legal representatives. Given their development of other template documents and guidance, it may be appropriate for the HRA to develop this template, utilising the findings from this study. Given the difficulties of representing the wishes and feelings of those whose wishes are unknown or could never be known, there may be a need to re-examine the legal basis for proxy decision-making in research for these populations.

## Conclusions

This study has examined the written information currently provided to family members, friends, and health and social care professionals involved in decisions about research on behalf of a person with impaired capacity. Existing study documents had ethical approval, yet many used inaccurate terms and lacked essential information, and some studies had incorrectly interpreted legal provisions. Particular issues were seen with the information provided to professionals acting as proxy in acute and critical care settings, where the clinical and representation roles were conflated. Whilst written information is usually accompanied by verbal explanation and supplementary information, the findings suggest that proxy decisions about research participation may not be sufficiently informed to meet the legal and ethical requirements.

Future research practice should focus on ensuring adequate information is provided to proxies in order for an informed decision to be made, thereby ensuring compliance with the legal frameworks. Further research is needed to explore the information and decision-making needs of those acting as proxies. There is a need to clarify the role of advance decisions and LPA in research and to re-examine the legal basis for decisions for those for whom there is no evidence of their wishes and feelings about research participation. These endeavours should focus on mechanisms for appropriate inclusion in research, and they should not be at the expense of further exclusion from research for these under-represented populations.

## Additional file

Additional file 1: Characteristics of included studies. (DOCX 18 kb)

## Abbreviations

ADRT: Advance Decision to Refuse Treatment
CTR: Medicines for Human Use (Clinical Trials) Regulations SI 2004/1031
LPA: Lasting Power of Attorney
MCA: Mental Capacity Act 2005
NIHR: National Institute for Health Research
UKCTG: UK Clinical Trials Gateway
