# Supplementary material for: Research involving adults lacking capacity to consent: a content analysis of participant information sheets for consultees and legal representatives in England and Wales
Source: Trials. 2019 Apr 25;20:233. doi: 10.1186/s13063-019-3340-5 (PMC6482489; doi:10.1186/s13063-019-3340-5)
Supplement: Supplementary file 1 — Characteristics of included studies. (DOCX 18 kb) [file 13063_2019_3340_MOESM1_ESM.docx]

**Additional file 1. Characteristics of included studies**

| **Study ID** | **Classification** | | **Recruiting countries** | | | **Funder** | | | **Sponsor** | | | **Population** |
| --- | --- | --- | --- | --- | --- | --- | --- | --- | --- | --- | --- | --- |
|  | **CTIMP** | **non-CTIMP** | **UK** | **EU** | **Other** | **NIHR** | **Charity** | **Other** | **HEI** | **NHS** | **Other** |  |
| 01 | X |  | X |  |  | X |  |  | X |  |  | learning disability |
| 02 |  | X | X |  |  | X |  |  | X |  |  | learning disability |
| 03 |  | X | X |  |  | X |  |  |  | X |  | learning disability |
| 04 |  | X | X |  |  | X |  |  | X |  |  | learning disability |
| 05 |  | X | X |  |  | X |  |  | X |  |  | care home residents |
| 06 |  | X | X |  |  | X |  |  | X |  |  | care home residents with dementia |
| 07 |  | X | X | X | X |  |  |  |  |  | X | progressive CNS disorder |
| 08 |  | X | X |  |  | X |  |  | X |  |  | care home residents |
| 09 | X |  | X |  |  | X |  |  |  | X |  | ventilated in ICU |
| 10 |  | X | X |  |  | X |  |  |  | X |  | people with dementia |
| 11 |  | X | X |  |  | X |  |  | X |  |  | care home residents with dementia |
| 12 |  | X | X |  |  | X |  |  |  | X |  | care home residents |
| 13 |  | X | X |  |  |  | X |  |  | X |  | care home residents |
| 14 |  | X | X |  |  | X |  |  | X |  |  | care home residents |
| 15 |  | X | X |  |  | X |  |  |  | X |  | care home residents |
| 16 |  | X | X |  |  | X |  |  | X |  |  | care home residents |
| 17 |  | X | X |  |  | X |  |  | X |  |  | ventilated in ICU |
| 18 |  | X | X | X | X | X |  |  | X | X |  | traumatic brain injury |
| 19 | X |  | X |  |  |  | X |  | X |  |  | traumatic haemorrhage |
| 20 | X |  | X | X |  | X |  |  | X |  |  | intracerebral haemorrhage |
| 21 | X |  | X | X | X |  | X |  | X |  |  | intracerebral haemorrhage or stroke |
| 22 |  | X | X |  |  |  | X |  |  | X |  | ventilated in ICU |
| 23 | X |  | X |  |  | X |  |  |  | X |  | ventilated in ICU |
| 24 | X |  | X |  |  |  | X | X | X |  |  | traumatic brain injury |
| 25 | X |  | X |  |  | X |  |  | X |  |  | acute neurological disorder |
| 26 |  | X | X |  |  |  | X |  |  | X |  | acute cardiac event |
| 27 | X |  | X | X |  | X | X |  | X |  |  | stroke |
| 28 |  | X | X |  |  |  |  | X | X |  |  | stroke |
| 29 |  | X | X |  |  | X |  |  | X |  |  | surgery |
| 30 |  | X | X |  |  | X |  |  |  | X |  | ventilated in ICU |

Key:

| CTIMP | Clinical trial of an investigational medicinal product - governed by CTR |
| --- | --- |
| Non-CTIMP | Research other than a clinical trial of an investigational medicinal product - governed by MCA |
| UK | United Kingdom (eligible if recruited participants in England and Wales, but may have recruited in other areas of UK) |
| EU | European Union |
| ICU | Intensive Care Unit |
| NIHR | National Institute for Health Research |
| HEI | Higher education institute (e.g university) |
| NHS | National Health Service provider |
| CNS | Central nervous system |
